# Prospects for improving the LHC W boson mass measurement with forward muons

**DOI:** 10.1140/epjc/s10052-015-3810-1

**Published:** 2015-12-17

**Authors:** Giuseppe Bozzi, Luca Citelli, Mika Vesterinen, Alessandro Vicini

**Affiliations:** Dipartimento di Fisica, Sezione di Milano, Università degli Studi di Milano and INFN, Via Celoria 16, 20133 Milan, Italy; Dipartimento di Fisica, Universita degli Studi di Milano, Via Celoria 16, 20133 Milan, Italy; Dipartimento di Fisica, Universita degli Studi di Milano and INFN Sezione di Milano and TIF Lab, Via Celoria 16, 20133 Milan, Italy; Physikalisches Institut, Ruprecht-Karls-Universitat, Im Neuenheimer Feld, 226, 69120 Heidelberg, Germany

## Abstract

Measurements of the *W* boson mass are planned by the ATLAS and CMS experiments, but for the time being, these may be unable to compete with the current world average precision of 15 MeV, due to uncertainties in the PDFs. We discuss the potential of a measurement by the LHCb experiment based on the charged lepton transverse momentum $p_T^{\ell }$ spectrum in $W \rightarrow \mu \nu$ decays. The unique forward acceptance of LHCb means that the PDF uncertainties would be anti-correlated with those of $p_T^{\ell }$ based measurements by ATLAS and CMS. We compute an average of ATLAS, CMS and LHCb measurements of $m_W$ from the $p_T^{\ell }$ distribution. Considering PDF uncertainties, this average is a factor of 1.3 more precise than an average of ATLAS and CMS alone. Despite the relatively low rate of *W* production in LHCb, we estimate that with the Run-II dataset, a measurement could be performed with sufficient experimental precision to exploit this anti-correlation in PDF uncertainties. The modelling of the lepton-pair transverse momentum distribution in the neutral current Drell–Yan process could be a limiting factor of this measurement and will deserve further studies.

## Introduction

The Standard Model precisely relates the mass of the *W* boson to the more precisely measured *Z* boson mass, fine structure constant and Fermi constant. The resulting indirect constraint on the *W* boson mass from a global fit [1–3] to experimental data is roughly a factor of two more precise than the direct measurement, $${m_{W}} = 80.385 \pm 0.015$$ GeV [4], leaving room for new physics, for example in supersymmetry [5]. The world average for $${m_{W}} $$ is dominated by measurements from the Fermilab Tevatron collider experiments, CDF [6, 7] and D0 [8, 9]. The CDF and D0 measurements used 2.1 and 4.9 fb$$^{-1}$$, respectively, out of the roughly 10 fb$$^{-1}$$ Tevatron Run-II dataset. Updates from both experiments are therefore highly anticipated. The current measurements are mostly limited by statistical uncertainties; either directly through limited *W* samples or indirectly through limited calibration samples. The uncertainty due to the parton distribution functions (PDFs) is around 10 MeV, with some variation between experiment, lepton flavour and fit variable. The Tevatron measurements used three different fit variables to extract $${m_{W}} $$:

1. The transverse mass, ${m_{T}^{W}} = \sqrt{2 {p_{T}^{\ell }} /\!\!\!\!E_{T}(1-\cos \phi )}$, where ${p_{T}^{\ell }}$ is the charged lepton transverse momentum, $/\!\!\!\!E_{T}$ is the missing transverse energy measured by the calorimeter, which estimates the neutrino transverse momentum; and $\phi$ is the azimuthal opening angle between the neutrino and charged lepton.
2. The charged lepton transverse momentum ${p_{T}^{\ell }}$ itself.
3. The missing transverse energy $/\!\!\!\!E_{T}$ itself.

The statistically most sensitive variable is $${m_{T}^{W}} $$, but with realistic $$/\!\!\!\!E_{T}$$ resolution, the $${p_{T}^{\ell }} $$ distribution has essentially the same sensitivity to $${m_{W}} $$. For example, in the CDF measurement [6, 7], the statistical uncertainties with the muon channel are 16 MeV and 18 MeV for the $${m_{T}^{W}} $$ and $${p_{T}^{\ell }} $$ fits, respectively.

Measurements are in progress by the ATLAS and CMS experiments at the LHC and the high pileup environment means that they may focus on $${p_{T}^{\ell }} $$ as their main fit variable  [10–12]. At the LHC, the *W* production cross section is roughly an order of magnitude higher than at the Tevatron, so the statistical uncertainties will retire from their dominant status. In the $$\sqrt{s}=1.96$$ TeV $$p\bar{p}$$ collisions at the Tevatron, *W* production was dominated by valence $$u\bar{d}$$ and $$d\bar{u}$$ annihilation. At the LHC, *W* production receives a larger contribution from sea quarks. Furthermore, the flavour composition is richer, with $$\mathcal {O}(20\,\%)$$ from $$c\bar{s}/s\bar{c}$$ annihilation. A $${m_{W}} $$ measurement at the LHC is therefore subject to potentially limiting PDF uncertainties. Ref. [13] casts a rather pessimistic outlook, while some recent studies are more optimistic [14–17]. Ref. [16] estimates an uncertainty of around 20 MeV using current PDF sets. However, this could be reduced to around 10 MeV with the requirement, $${p_{T}^W} < 15$$ GeV on the lepton-pair transverse momentum; this cut makes the shape of the charged-lepton transverse momentum distribution steeper and closer to the leading-order one; it also suppresses the contribution from *qg* scattering. The studies reported in this paper assume that ATLAS and CMS will make this requirement in their measurements. It is also pointed out in Ref. [16] that the uncertainty would be greatly reduced if the pseudorapidity, $$\eta $$, acceptance could be extended from the roughly $$|\eta | < 2.5$$ of ATLAS and CMS, to $$|\eta | < 4.9$$, because of an anti-correlation between the parton-parton luminosities and the lepton transverse momentum distribution at different charged lepton rapidities. Ref. [17] proposed that even within the limited acceptance of the ATLAS and CMS detectors, an exploitation of the correlations between different rapidity regions and with the two *W* charges could reduce the uncertainty by around 60 %. Further improvements could be achieved by exploiting the correlations with $${Z/\gamma ^*} $$ decays [17].

So far it has been assumed that ATLAS and CMS are the only LHC experiments with a chance to improve on the direct $${m_{W}} $$ determination. The LHCb experiment [18] has not been discussed in this context.

- Firstly, the rate of *W* production is far smaller in LHCb due to (i) the limited angular acceptance $2 < \eta < 5$ and (ii) the lower instantaneous luminosity.^1^
- Secondly, LHCb lacks the hermetic calorimeter coverage that is needed to reconstruct the $$/\!\!\!\!E_{T}$$ and $${m_{T}^{W}} $$. The only available observable is thus $${p_{T}^{\ell }} $$.

In this paper, we argue that *W* production in LHCb is sufficient to make a competitive measurement, using the $${p_{T}^{\ell }} $$ distribution, and quantify the sensitivity with current and future datasets. The unique angular acceptance of LHCb turns out to be a complement to the ATLAS and CMS measurements when we consider the PDF uncertainties. In fact, the ability for LHCb to select a pure sample of $$W \rightarrow \mu \nu $$ decays without any requirement on the $$/\!\!\!\!E_{T}$$, as already demonstrated in [19], is likely to be an advantage. A key challenge in the Tevatron $${m_{W}} $$ measurements was the calibration of the detector response to the hadronic recoil. This would be completely avoided in the LHCb measurement, which essentially only requires knowledge of the muon reconstruction.^2^ The present study has been performed assuming a given production model of the *W* boson, i.e. making definite choices for the description of the QCD corrections that affect the $${p_{T}^{\ell }} $$ distribution.

In Sect. 2 the study of PDF uncertainties reported in Ref. [16] is extended to consider the impact of a LHCb measurement. In Sect. 3, the expected experimental uncertainties on a $${m_{W}} $$ measurement are estimated. We choose to focus on the data that will be collected during Run-II (2015–2018) at a centre of mass energy of $$\sqrt{s} = 13$$ TeV. It is expected that LHCb will record at least 7 fb$$^{-1}$$. The prospects for a LHC combination are discussed in Sect. 4. In Sect.5 we comment on the uncertainties stemming from the $${p_{T}^W} $$ modelling.

## PDF uncertainties

Our analysis is based on exactly the same setup as in Ref. [16]. Events of the type $$pp \rightarrow W \rightarrow \ell \nu +X$$, at $$\sqrt{s}=13$$ TeV, are generated using POWHEG [20], with parton showering provided by PYTHIA [21]. Replica templates for the $${p_{T}^{\ell }} $$ distribution are produced for each of the NNPDF3.0 [22], MMHT2014 [23] and CT10 [24] PDF sets. For the sake of simplicity, we assume the same kinematic acceptance for the ATLAS and CMS experiments, and henceforth refer to them generically as the General Purpose Detector (GPD) experiments. The GPD acceptance is defined as; $$|\eta | < 2.5$$, $${p_{T}^{\ell }} > 25$$ GeV, $${p_{T}^{\nu }} > 25$$ GeV, $${p_{T}^W} < 15$$ GeV.^3^ For LHCb, the kinematic acceptance is defined to be $$2.0 < \eta < 4.5$$ and $${p_{T}^{\ell }} > 20$$ GeV. The possibility of cut on $${p_{T}^{\nu }} $$ and/or $${p_{T}^W} $$ is obviously excluded for LHCb. For simplicity, we assume a GPD averaged measurement for each *W* charge, already averaged over electron and muon channels. In the following, these are denoted $$\mathbf {G^+}$$ and $$\mathbf {G^-}$$. The two LHCb measurements with $$W \rightarrow \mu \nu $$ are denoted $$\mathbf {L^+}$$ and $$\mathbf {L^-}$$.

We follow the PDF4LHC recommendation [25] in estimating the PDF uncertainty. If we consider the three sets (NNPDF3.0, MMHT2014, and CT10), then the full uncertainty envelope of the considered sets is used. In our default evaluation, we only consider the two most recent sets (NNPDF3.0 and MMHT2014), which already include constraints from LHC data. The following uncertainties (in MeV) are estimated:

1 $$\begin{aligned} {\delta _\mathrm{PDF}} = {}\left( \begin{array}{c@{\quad }c}{\mathbf {G^+}} &{} 24.8\\ {\mathbf {G^-}} &{} 13.2\\ {\mathbf {L^+}} &{} 27.0\\ {\mathbf {L^-}} &{} 49.3\\ \end{array} \right) , \end{aligned}$$

These are repeated in Table 1, while Table 2 lists the corresponding uncertainties that are evaluated with the inclusion of the CT10 sets. In both tables, we also provide the largest difference in central values, denoted $\Delta _\mathrm{sets}$, between the (two or three) sets under consideration in each case. This is evidently a major contributor to the uncertainty envelope. For the $W^+$, similar uncertainties are estimated for LHCb and the GPDs. For the $W^-$ on the other hand, the LHCb uncertainty is roughly a factor of four larger, because of the uncertainty of the down valence quark (the $d\bar{u}$ induced subprocess gives the largest contribution to the cross section) together with the large uncertainty of the sea quarks at large partonic *x*. The real power of the LHCb measurement is revealed in the correlations. With the NNPDF3.0 sets, we obtain the following correlation matrix:

2 $$\begin{aligned} \rho = {}\left( \begin{array}{c@{\quad }c@{\quad }c@{\quad }c@{\quad }c}&{} \mathbf {G^{+}}&{} \mathbf {G^{-}}&{} \mathbf {L^{+}}&{} \mathbf {L^{-}}\\ \mathbf {G^{+}} &{} 1&{}&{}&{}\\ \mathbf {G^{-}} &{} -0.22&{}1&{}&{}\\ \mathbf {L^{+}} &{} -0.63&{}0.11&{}1&{}\\ \mathbf {L^{-}} &{} -0.02&{}-0.30&{}0.21&{}1\\ \end{array} \right) . \end{aligned}$$

**Fig. 1:**
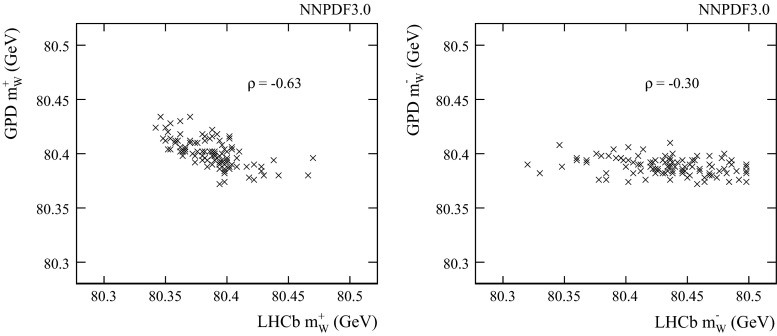
The fitted $${m_{W}} $$ in the GPDs versus LHCb for each NNPDF3.0 set, and for (*left*) $$W^+$$ and (*right*) $$W^-$$.eps

**Fig. 2:**
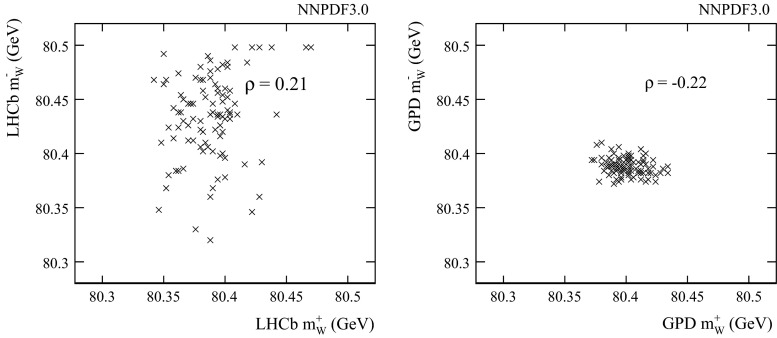
The fitted $${m_{W}} $$ for $$W^+$$ versus $$W^-$$ and for (*left*) LHCb and (*right*) the GPDs. Based in the NNPDF3.0 PDF sets

There is a particularly large negative correlation of around $$-60$$ % between the LHCb and GPD measurements with the $$W^+$$, and a smaller anti-correlation of around $$-30$$ % for the $$W^-$$. Similar correlation coefficients are found with the two other sets under consideration.^4^ This can be clearly seen in Fig. 1 which shows the distribution of fitted $${m_{W}} $$ values in the GPDs versus LHCb for the 100 NNPDF3.0 replicas. For a single experiment, there are smaller correlations between the $$W^+$$ and $$W^-$$ measurements, as can be seen in Fig. 2. In LHCb, this is around $$+20$$ %, and for the GPDs, it is around $$-20$$ %. Between different charges and experiments, the correlations are around 10 % or less in magnitude. The normalised set of weights $$\alpha _i$$ that minimises the uncertainty on the weighted average of the four measurements $${m_{W}} _i$$,

3 $$\begin{aligned} {m_{W}} = \sum \limits _{i=1}^4 \alpha _i {m_{W}} _i, \end{aligned}$$

would be

4 $$\begin{aligned} \alpha = {}\left( \begin{array}{c@{\quad }c}{\mathbf {G}}^{+} &{} 0.30\\ {\mathbf {G}}^{-} &{} 0.45\\ {\mathbf {L}}^{+} &{} 0.21\\ {\mathbf {L}}^{-} &{} 0.04\\ \end{array} \right) \end{aligned}$$

The resulting PDF uncertainty would be 10.5 MeV with the GPDs alone, and 7.7 MeV including LHCb. Table 3 lists the PDF uncertainties, with and without including LHCb. The set of weights is also listed. An average that includes ${\mathbf {L^+}}$ with around 20 % of the weight, and with only a few percent for ${\mathbf {L^-}}$, would have a PDF uncertainty that is reduced by more than 30 %. Table 3 also lists the corresponding numbers for scenarios in which:

- The CT10 sets are included in the uncertainty estimates: In this case the PDF uncertainties are increased by roughly a factor of two, but the relative impact of the LHCb measurement is similar to the 2-set scenario.
- Each PDF set is considered separately: instead of the envelope, the individual uncertainty bands are used. The uncertainties are far smaller, but LHCb still has a large impact. For the NNPDF3.0 sets, the gain is still around 30 %. For the other two sets, the gain is closer to a factor of two!

The next question is whether or not LHCb can measure $${m_{W}} $$ with sufficient experimental precision to exploit this anti-correlation in PDF uncertainties.

**Table 1:** PDF uncertainties on $${m_{W}} $$ (MeV) with the PDF4LHC prescription using the NNPDF3.0 and MMHT2014 sets, for the 4 sub-measurements as defined in the text

	${\mathbf {G^+}}$	${\mathbf {G^-}}$	${\mathbf {L^+}}$	${\mathbf {L^-}}$
Envelope	24.8	13.2	27.0	49.3
$\Delta _\mathrm{sets}$	20.9	5.7	12.1	22.9

**Table 2:** PDF uncertainties on $${m_{W}} $$ (MeV) with the PDF4LHC prescription using the NNPDF3.0, MMHT2014 and CT10 sets, for the 4 sub-measurements as defined in the text

	${\mathbf {G^+}}$	${\mathbf {G^-}}$	${\mathbf {L^+}}$	${\mathbf {L^-}}$
Envelope	29.9	23.5	35.0	84.1
$\Delta _\mathrm{sets}$	22.0	23.7	24.0	74.0

**Table 3:** The PDF uncertainties on the LHC averages including and excluding LHCb, resulting from the weighted average with the optimal weights, $\alpha$

PDFs	Experiments	${\delta _\mathrm{PDF}}$ (MeV)	$\alpha$
PDF4LHC(2-sets)	2 $\times$ GPD	10.5	(0.26, 0.74, 0, 0)
PDF4LHC(2-sets)	2 $\times$ GPD + LHCb	7.7	(0.30, 0.45, 0.21, 0.04)
PDF4LHC(3-sets)	2 $\times$ GPD	16.9	(0.50, 0.50, 0, 0)
PDF4LHC(3-sets)	2 $\times$ GPD + LHCb	12.7	(0.43, 0.41, 0.11, 0.04)
NNPDF30	2 $\times$ GPD	5.2	(0.50, 0.50, 0, 0)
NNPDF30	2 $\times$ GPD + LHCb	3.6	(0.35, 0.47, 0.16, 0.02)
MMHT2014	2 $\times$ GPD	9.2	(0.45, 0.55, 0, 0)
MMHT2014	2 $\times$ GPD + LHCb	4.6	(0.39, 0.14, 0.46, 0)
CT10	2 $\times$ GPD	11.6	(0.33, 0.67, 0, 0)
CT10	2 $\times$ GPD + LHCb	6.3	(0.38, 0.20, 0.40, 0.03)

## LHCb experimental sensitivity to the W mass

In Run-I (2010–2012), LHCb recorded 3 fb$$^{-1}$$ of *pp* collisions at $$\sqrt{s}=7-8$$ TeV. In Run-II (2015–2018), LHCb hopes to record around 7 fb$$^{-1}$$ at $$\sqrt{s}=13$$ TeV. Given the $$W \rightarrow \mu \nu $$ signal yields reported in a LHCb measurement using only 1 fb$$^{-1}$$ of data from the 2011 Run [26], we extrapolate the projected Run-I and Run-II signal yields, and use these to estimate the uncertainties on a $${m_{W}} $$ measurement with LHCb. These estimates are listed in Table 4, for both the Run-I and Run-II datasets. They are quoted separately for the $$W^+$$ and $$W^-$$ since the PDF uncertainties, as discussed in detail in Sect. 2, motivate separate analyses for the two charges.

**Table 4:** The estimated experimental uncertainties on a $${m_{W}} $$ measurement with LHCb

	Run-I		Run-II	
	3 fb$^{-1}$		7 fb$^{-1}$	
	$W^+$	$W^-$	$W^+$	$W^-$
Signal yields, $\times 10^{6}$	1.2	0.7	5.4	3.4
$Z/\gamma ^*$ background, (*B* / *S*)	0.15	0.15	0.15	0.15
QCD background, (*B* / *S*)	0.15	0.15	0.15	0.15
$$\delta _{{m_{W}}}$$ (MeV)				
Statistical	19	29	9	12
Momentum scale	7	7	4	4
Quadrature sum	20	30	10	13

### Statistical sensitivity estimate for the ${p_{T}^{\ell }}$ fit

In Ref. [26], LHCb found, in 1 fb$^{-1}$ of Run-I data, around 550k candidate muonic $W^+$ decays, and around 350k $W^-$, with a purity of around 70 %. The extrapolated signal yields in the full Run-I and Run-II datasets are listed in Table 4. The cross sections for $W^{\pm }$ production increase by a factor close to two when the collision energy increases from 7 to 13 TeV.^5^ The Run-I yield of around two million can be compared with the 0.6(0.5) million $W \rightarrow \mu (e)\nu$ candidates that were used in the CDF measurement with 2.1 fb$^{-1}$ [6, 7]. The D0 measurement with 4.3 fb$^{-1}$ [8, 9] used around 1.7 million $W \rightarrow e\nu$ signal candidates. The Run-II $W \rightarrow \mu \nu$ yield in LHCb, assuming an integrated luminosity of 7 fb$^{-1}$, will be around eight million.

**Fig. 3:**
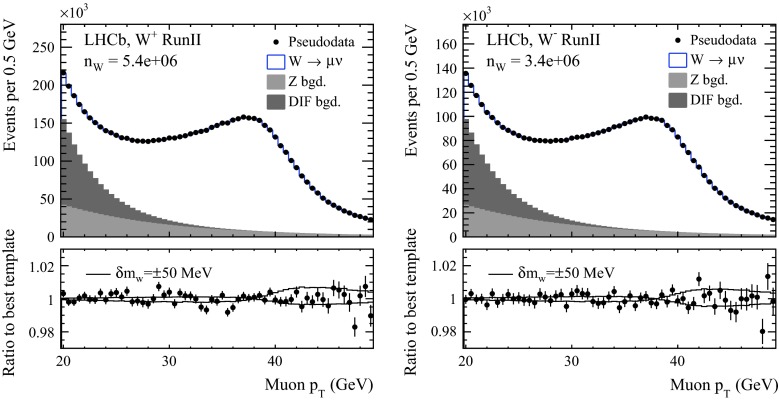
The simulated $${p_{T}^{\ell }} $$ spectra for (*left*) $$W^+$$ and (*right*) $$W^-$$ decays in the projected Run-II LHCb dataset. The data points correspond to one of the 500 pseudo-datasets. The stacked histogram corresponds to the best fit template. In the *lower panel*, the points represent the ratio of the data to the best fit template, and the *lines* show the ratio of the best fit template to templates with $${m_{W}} $$ varying by $$\pm 50$$ MeV

In order to estimate the statistical precision on the $${m_{W}} $$ fit with LHCb data, we take the $${p_{T}^{\ell }} $$ templates described in Sect. 2. The dominant background reported in Ref. [26] is $$Z/\gamma ^* \rightarrow \mu \mu $$ where one muon escapes the limited angular acceptance of LHCb. At lower $${p_{T}^{\ell }} $$, there is a large “QCD” background from muonic decays of pions and kaons. Directly under the upper edge Jacobian peak, where most of the $${m_{W}} $$ sensitivity is delivered, the QCD background is small. An exponential parameterisation for each of the $${Z/\gamma ^*} $$ and QCD backgrounds is added to the signal $${p_{T}^{\ell }} $$ templates, with yields and shapes roughly reproducing those in Ref. [26]. The signal and background templates are scaled to the projected yields listed in Table 4. From this spectrum, we generate 500 copies but with each bin varied according to a Poisson random number. Each of the 500 pseudo-datasets is compared to the ensemble of templates corresponding to different $${m_{W}} $$ values. An example of these pseudo-datasets is compared in Fig. 3 to the sum of signal and background templates for one $${m_{W}} $$ hypothesis. These are shown separately for the two *W* charges, and in each case, the lower section of the figure shows the ratio of the data points to the best fit template, and to the templates corresponding to shifts of $$\pm 50$$ MeV in $${m_{W}} $$. The best fit value for each of these 500 experiments is obtained from the minimum $$\chi ^2$$ and with the uncertainty defined by $$\Delta \chi ^2 = \pm 1$$. Table 4 lists the statistical uncertainty computed as the spread of the best fit central values.^6^ With the Run-II dataset, LHCb could achieve statistical uncertainties of 10(13) MeV for the $$W^+$$($$W^-$$).

### Momentum scale calibration

In the Tevatron $${m_{W}} $$ measurements, the muon momentum scale, and electron energy scale, were major contributors to the total uncertainties on $${m_{W}} $$. In particular, the D0 measurement with $$W \rightarrow e\nu $$ relied almost entirely on $$Z/\gamma ^* \rightarrow e^+e^-$$ events, leading to the single largest source of uncertainty of around 20 MeV, depending on the fit variable. The CDF measurement exploited a combination of $$J/\psi $$, $$\psi (2S)$$, $$\Upsilon (nS)$$ ($$n=1,2,3$$) and $$Z/\gamma ^*$$ decays into $$\mu ^+\mu ^-$$ to achieve a muon momentum scale uncertainty of 7 MeV.

LHCb is ideally suited for a precise calibration of the momentum scale, due to the large samples of inclusive quarkonia signals that are recorded. Furthermore, LHCb has an excellent momentum resolution that ranges between 0.2 and 0.8 % [18]. LHCb has already demonstrated its ability to make world’s best measurements of various *b*- and *c*-hadron masses [27, 28]. In Ref. [27], LHCb reported a relative momentum scale uncertainty of $3 \times 10^{-4}$ as part of a measurement of *b*-baryon masses, using only 35 pb$^{-1}$ of data.

The $$Z/\gamma ^* \rightarrow \mu ^+\mu ^-$$ line shape provides an important constraint on the momentum scale at high $$p_{T}$$. Roughly speaking this would be $$\delta _z/\sqrt{N}$$, where *N* is the number of $${Z/\gamma ^*} $$ events in the *Z* peak region, and the observed line-width, $$\delta _z \sim 3$$ GeV, is dominated by the natural width of the *Z*. A concern might be that while LHCb records enough *W* decays, the limited angular acceptance might not allow sufficient $${Z/\gamma ^*} $$ samples. In Ref. [29], LHCb found, in 1 fb$$^{-1}$$ of Run-I data, around 60 k $$Z/\gamma ^* \rightarrow \mu \mu $$ candidates with almost perfect purity. We estimate around 150k signal in the full Run-I dataset and around 700k in Run-II, yielding naive momentum scale uncertainties of 7 MeV and 3 MeV, respectively. Further constraints can be obtained from the $$J/\psi $$ and $$\Upsilon $$ resonances. Extrapolating the $$\Upsilon (1S)$$ yields reported in [30], the full Run-I dataset should already provide a few million decays. A dedicated study would be needed to demonstrate that the alignment of the LHCb tracking detectors could be understood with sufficient precision to relate these mass constraints to the momentum scale. For the purpose of our present study, we assume a momentum scale uncertainty on $${m_{W}} $$ of 7(4) MeV for the Run-I(II) datasets.

### Muon efficiencies

The methods to measure muon reconstruction efficiencies in LHCb are well developed as part of the $$Z/\gamma ^*$$ cross section measurements [19]. Since the $${m_{W}} $$ measurement is only concerned with the kinematic dependence of the efficiency and not in its absolute scale, it can safely be assumed that this will be a sub-dominant source of uncertainty.

## Prospects for an LHC $${m_{W}} $$ combination

The experimental precision with which ATLAS and CMS can measure $${m_{W}} $$ will no doubt have evolved since the discussions in Refs. [10, 11]. The idea of this study is not to make a precise estimate of the LHC sensitivity, but rather to estimate the relative impact of the proposed LHCb measurement. Our assumption is that ATLAS and CMS can both measure $${m_{W}} $$ with experimental uncertainties of ±7 MeV per *W* charge, having averaged over electron and muon decay channels. Large variations either side of this assumption are considered in our study.

The four measurements would have the following uncertainties, using the NNPDF3.0 and MMHT2014 PDF sets,

5 $$\begin{aligned} \delta m_W^i = \left( \begin{array}{c@{\quad }l} \mathbf {G^+} &{}(7_\mathrm{exp} \pm 25_\mathrm{PDF})~\mathrm {MeV}\\ \mathbf {G^-} &{}(7_\mathrm{exp} \pm 13_\mathrm{PDF})~\mathrm {MeV}\\ \mathbf {L^+} &{}(10_\mathrm{exp} \pm 27_\mathrm{PDF})~\mathrm {MeV}\\ \mathbf {L^-} &{}(13_\mathrm{exp} \pm 49_\mathrm{PDF})~\mathrm {MeV}\\ \end{array} \right) . \end{aligned}$$

For the sake of simplicity, our study only considers experimental and PDF errors, while for a more realistic estimate one should include also other sources of theoretical uncertainty. For each experiment, we assume a positive correlation of 50 % between the experimental uncertainties for $W^+$ and $W^-$, as can be expected since many experimental calibrations are independent of the charge. The set of weights are optimised to give the smallest total uncertainty on the weighted average of the four measurements. The resulting uncertainties and optimal weights are listed in Table 5. The first three rows show the results of (i) the LHC average including all three experiments with muons and electrons (only for the GPDs) and both charges, (ii) a combination of LHCb and one GPD, (iii) a combination of the two GPDs without LHCb. The total uncertainty is improved by around 30 % when LHCb is included. Interestingly, an average of LHCb with a single GPD would be more precise than a two-GPD combination. Table 5 also lists the corresponding uncertainties and weights for a number of variations in our assumptions.

- If all three PDF sets are used with the PDF4LHC prescription, the total uncertainty is larger, and the impact of LHCb is even larger than with the two more recent sets.
- We consider the four possibilities of setting the LHCb or GPD experimental uncertainties to zero or twice our nominal assumption. In all cases, LHCb is more important in the average, than a second GPD.
- Not surprisingly, LHCb has more(less) impact if we scale the PDF uncertainties by a factor of two(zero).

It seems that in any realistic scenario, excluding the extreme cases above, LHCb would reduce the total uncertainty on the LHC average by 20–40 %. And in all of these scenarios, we have the remarkable result that LHCb has more impact than a second GPD.

**Table 5:** The uncertainties on different LHC averages for $${m_{W}} $$. The separate experimental and PDF uncertainties are listed, as are the weights that minimise the total uncertainty

Scenario	Experiments	$\delta m_W$ (MeV)			
		Tot	Exp	PDF	$\alpha$
Default	2 $\times$ GPD + LHCb	9.0	4.7	7.7	(0.30, 0.44, 0.22, 0.04)
Default	1 $\times$ GPD + LHCb	10.1	6.5	7.7	(0.31, 0.40, 0.25, 0.04)
Default	2 $\times$ GPD	12.0	5.8	10.5	(0.28, 0.72, 0, 0)
PDF4LHC(3-sets)	2 $\times$ GPD + LHCb	13.6	4.8	12.7	(0.43, 0.41, 0.12, 0.04)
PDF4LHC(3-sets)	1 $\times$ GPD + LHCb	14.6	7.3	12.7	(0.43, 0.40, 0.12, 0.04)
PDF4LHC(3-sets)	2 $\times$ GPD	17.7	5.5	16.9	(0.50, 0.50, 0, 0)
$\delta _\mathrm{exp}^\mathrm{LHCb} = 0$	2 $\times$ GPD + LHCb	8.7	4.0	7.7	(0.31, 0.41, 0.24, 0.04)
$\delta _\mathrm{exp}^\mathrm{LHCb} = 0$	1 $\times$ GPD + LHCb	9.8	5.9	7.9	(0.31, 0.37, 0.28, 0.04)
$\delta _\mathrm{exp}^\mathrm{LHCb} = 0$	2 $\times$ GPD	12.0	5.8	10.5	(0.28, 0.72, 0, 0)
$\delta _\mathrm{exp}^\mathrm{GPD} = 0$	2 $\times$ GPD + LHCb	7.9	1.9	7.7	(0.29, 0.48, 0.19, 0.04)
$\delta _\mathrm{exp}^\mathrm{GPD} = 0$	1 $\times$ GPD + LHCb	7.9	1.9	7.7	(0.29, 0.48, 0.19, 0.04)
$\delta _\mathrm{exp}^\mathrm{GPD} = 0$	2 $\times$ GPD	10.5	0.1	10.5	(0.26, 0.74, 0, 0)
$\delta _\mathrm{PDF} = 0$	2 $\times$ GPD + LHCb	4.6	4.6	0.0	(0.34, 0.34, 0.22, 0.10)
$\delta _\mathrm{PDF} = 0$	1 $\times$ GPD + LHCb	5.8	5.8	0.0	(0.23, 0.23, 0.37, 0.17)
$\delta _\mathrm{PDF} = 0$	2 $\times$ GPD	5.5	5.5	0.0	(0.50, 0.50, 0, 0)
$\delta _\mathrm{exp}^\mathrm{LHCb} \times 2$	2 $\times$ GPD + LHCb	9.6	5.6	7.7	(0.29, 0.50, 0.17, 0.04)
$\delta _\mathrm{exp}^\mathrm{LHCb} \times 2$	1 $\times$ GPD + LHCb	10.8	7.6	7.7	(0.30, 0.46, 0.20, 0.05)
$\delta _\mathrm{exp}^\mathrm{LHCb} \times 2$	2 $\times$ GPD	12.0	5.8	10.5	(0.28, 0.72, 0, 0)
$\delta _\mathrm{exp}^\mathrm{GPD} \times 2$	2 $\times$ GPD + LHCb	11.2	7.9	8.0	(0.32, 0.35, 0.29, 0.04)
$\delta _\mathrm{exp}^\mathrm{GPD} \times 2$	1 $\times$ GPD + LHCb	13.9	10.5	9.0	(0.31, 0.26, 0.37, 0.05)
$\delta _\mathrm{exp}^\mathrm{GPD} \times 2$	2 $\times$ GPD	15.6	11.5	10.6	(0.32, 0.68, 0, 0)
$\delta _\mathrm{PDF} \times 2$	2 $\times$ GPD + LHCb	16.0	4.7	15.3	(0.30, 0.45, 0.21, 0.04)
$\delta _\mathrm{PDF} \times 2$	1 $\times$ GPD + LHCb	16.7	6.7	15.3	(0.30, 0.44, 0.22, 0.04)
$\delta _\mathrm{PDF} \times 2$	2 $\times$ GPD	21.7	5.9	20.9	(0.27, 0.73, 0, 0)

## Uncertainties stemming from the $${p_{T}^W} $$ modelling

Another source of theoretical uncertainty that we have overlooked so far is the $${p_{T}^W} $$ model. This strongly affects the preparation of the templates that are used to fit the data and eventually to extract $${m_{W}} $$. The presence, at low lepton-pair transverse momenta, of large logarithmically enhanced QCD corrections and the role, in the same kinematic region, of non-perturbative effects have been discussed in Refs. [31, 32], but the dependence of the resulting model on the acceptance cuts has never been investigated in detail and will deserve a dedicated study. The $${p_{T}^{\ell }} $$ is more sensitive to this than $${m_{T}^{W}} $$ . At the Tevatron, the uncertainty from the $${p_{T}^W} $$ model on the $${p_{T}^{\ell }} $$ fit was around 5 MeV, but perturbative QCD scale uncertainties should also be taken into account. To a first approximation the results of the present note are independent of the uncertainty stemming from on the $${p_{T}^W} $$ modelling and will hopefully be confirmed if the latter will turn out to be under control. On a longer term perspective we will need a global analysis of all the non-perturbative elements active in the proton description: the PDFs uncertainties, in particular the role of heavy quarks in the proton [12, 33], and the description of the intrinsic transverse momentum of the partons. The inclusion of all the different Drell–Yan channels (neutral current, $$W^+$$ and $$W^-$$) in the different acceptance regions of the LHC experiments might have an impact on a systematic reduction of all these uncertainties.

## Summary

Improving the precision on $${m_{W}} $$ remains a priority in particle physics. At the LHC, there is no shortage of *W* production, but there are potentially limiting PDF uncertainties on the anticipated measurements by ATLAS and CMS, which cover central lepton pseudorapidities, $$|\eta | \lesssim 2.5$$. We show that a measurement in the forward acceptance of the LHCb experiment, $$2 < \eta < 4.5$$, would have a PDF uncertainty that is highly anti-correlated with those of ATLAS and CMS. In this paper we study the measurement of $${m_{W}} $$ extracted from the $${p_{T}^{\ell }} $$ distribution. The weighted average of the ATLAS, CMS and LHCb results, based only on the PDF uncertainties, would be 30 % more precise than an average of ATLAS and CMS alone. Despite the lower rate of *W* production in LHCb, a measurement could be made with the Run-II dataset, using $$W \rightarrow \mu \nu $$ decays, that improves the total uncertainty on the LHC average by 20–40 %, depending on the assumptions on the experimental uncertainties. In fact, for any realistic scenario, LHCb has more impact in the LHC average than a second GPD. It remains to be demonstrated that the $${p_{T}^W} $$ model uncertainties can be controlled at the necessary level of precision, but deeper study into a possible $${m_{W}} $$ measurement with LHCb, and its combination with the ATLAS and CMS measurements, is well motivated.

## Footnotes

1 In 2012, LHCb already received a factor of twenty lower instantaneous luminosity than ATLAS and CMS, as required for the beauty and charm physics program.

2 It should be noted that current LHCb studies of *W* production have imposed tight isolation requirements on the muon [19], the efficiency of which has some sensitivity to the hadronic recoil model.

3 We assume that the GPD experiments will adopt the suggestion of Ref. [16], to require ${p_{T}^W} < 15$ GeV.

4 The CT10 sets yield coefficients of $-72$ and $-21$ % for the $W^+$ and $W^-$, respectively. With the MMHT2014 sets, the corresponding coefficients are $-67$ and $-24$ %.

5 The leading order cross section increases by a factor 2.3 in the $W^+$ case and by 2.6 in the $W^-$ case, when changing the collision energy from 7 to 13 TeV. We assume a factor of two in order to be conservative.

6 The $\pm 29$ MeV uncertainty for the $W^-$ in Run-I can be compared to the $\pm 22$ MeV that was reported by CDF in the ${p_{T}^{\ell }}$ fit using a similar number of $W\rightarrow \mu \nu$ events [6, 7].
